# Risk consciousness and public perceptions of COVID-19 vaccine passports

**DOI:** 10.1177/05390184231182056

**Published:** 2023-06-21

**Authors:** Btihaj Ajana, Elena Engstler, Anas Ismail, Marina Kousta

**Affiliations:** King’s College London, UK; King’s College London, UK; King’s College London, UK; King’s College London, UK

**Keywords:** COVID-19, health, public perceptions, risk, Ulrich Beck, vaccine passports, COVID-19, passeports vaccinaux, perceptions du public, risque, santé, Ulrich Beck

## Abstract

In response to the global outbreak of COVID-19 in early 2020, many countries around the world have rushed to develop and implement various mechanisms, including vaccination passports, to contain the spread of the virus and manage its significant impact on heath and society. COVID-19 passports have been promoted as a way of speeding society’s return to ‘normal’ life while protecting public health and safety. These passports, however, are not without controversy. Various concerns have been raised with regard to their social and ethical implications. Framing the discussion within the ‘risk society’ thesis and drawing on an interview-based study with members of the UK public as well as the relevant literature, this article examines perceptions of COVID-19 vaccine passports. The findings of the study indicate that participants’ attitudes toward vaccine passports are primarily driven by factors relating to perceptions of risk. While some considered vaccine passports as a positive strategy to encourage vaccine uptake and facilitate travel and daily activities, others saw this mechanism as a coercive step that might alienate further those who are already vaccine hesitant. Issues of fairness, equity, discrimination, trust, and data security were major themes in participants’ narratives and their subjective assessment of vaccine passports.

## Résumé

En réponse à l’épidémie mondiale de COVID-19 au début de l’année 2020, de nombreux pays dans le monde se sont empressés d’élaborer et de mettre en œuvre divers mécanismes, dont les passeports vaccinaux, afin de contenir la propagation du virus et de gérer son impact considérable sur la santé et la société. Les passeports COVID-19 ont été présentés comme un moyen d’accélérer le retour de la société à la vie « normale » tout en protégeant la santé et la sécurité publiques. Ces passeports ne vont toutefois pas sans controverse. Leurs implications sociales et éthiques ont suscité de nombreuses inquiétudes. En inscrivant la discussion dans le cadre de la thèse de la « société du risque » et en s’appuyant sur une étude basée sur des entretiens avec des membres du public britannique ainsi que sur la littérature pertinente, cet article examine les perceptions des passeports vaccinaux de COVID-19. Les résultats de l’étude indiquent que les attitudes des participants à l’égard des passeports vaccinaux sont principalement motivées par des facteurs liés à la perception du risque. Alors que certains considèrent le passeport vaccinal comme une stratégie positive pour encourager la vaccination et faciliter les voyages et les activités quotidiennes, d’autres considèrent ce mécanisme comme une mesure coercitive qui pourrait aliéner davantage ceux qui hésitent déjà à se faire vacciner. Les questions de justice, d’équité, de discrimination, de confiance et de sécurité des données ont été des thèmes majeurs dans les récits des participants et dans leur évaluation subjective des passeports vaccinaux.

## Introduction and conceptual background

The outbreak of the coronavirus disease (COVID-19) in early 2020 has led to the development and deployment of various solutions to manage and contain the spread of this virus. Along with the rollout of COVID-19 vaccines came myriad debates and propositions about the need to introduce vaccination passports as a means of enabling the return of society to a sense of normality. Serving as a proof of COVID-19 vaccine status, vaccination passports have been deployed in many countries around the world during the current pandemic in order to safely ease COVID-19-related restrictions, facilitating the return to work, access to public venues and travel, and generally, enabling the resumption of other aspects of daily life that have been hindered by the pandemic.

As mentioned in a report by the Ada Lovelace Institute (2021a), there has been a confusion with regard to the different terms used to describe COVID-19 vaccine passports. Also referred to as immunity or immunization passports, or COVID-19 status certificates, these artifacts are accredited documents that share the common function of linking health status (immunity/vaccine status and/or test results) with verification of identity. Whereas immunity passports tend to certify that the person got infected with and recovered from the disease, vaccination passports certify that the person received the vaccine against the disease. Thus, the overarching aim of such passports and certificates is to determine whether an individual has had a COVID-19 vaccine or has recently recovered from the virus. This information can, in turn, determine the permissions and rights this individual can have, such as access to travel, work, and leisure spaces. Although these artifacts can be in paper or digital form, most of the debates on vaccine passports have so far focused on the digital format, looking, for instance, at the implications of the data-driven infrastructures and blockchain technologies that have been proposed or deployed to manage the nexus of identity and health status. In this article, we also focus on the digital COVID-19 vaccine passports.

Ostensibly, the concept of vaccine passports or immunity certificates is not a new invention nor is it confined to the context of COVID-19 pandemic. The meningitis, polio, and yellow fever certificates are examples of the vaccination documents that have been in use globally way before the outbreak of COVID-19. But what differentiates such certificates from COVID-19 passports is that they are used merely for travel purposes across borders. COVID-19 passports/certificates are, instead, meant to manage not only cross-country travel but also domestic travel and access to everyday spaces and activities, including restaurants and cafés, gyms and nightclubs, public transport, and so on. They are, as such, ‘technologies of the everyday’ that rely on digital solutions to manage the risk of COVID-19 at the individual level, allowing or limiting movement and access, accordingly. Given their nature, function, and purpose, it is not surprising that COVID-19 vaccine passports have sparked a lot of controversy, as many issues have been raised within policy debates, news reports, and the academic literature alike regarding the feasibility and socio-ethical implications of these artifacts of pandemic governance.

In this article, we report on the findings of an interview-based study we conducted with members of the UK public, looking at lay people’s perceptions of COVID-19 vaccine passports and to what extent such perceptions are shaped by understandings and constructions of risk regarding vaccine passports. Risk is indeed a crucial concept in the name of which many policies and responses have been mobilized to contain coronavirus, producing along the way categories of the ‘risky’ and the ‘at risk’ (Cook et al., 2021: 210) whose conduct and safety are being managed through various techniques and technologies. As Lupton (2022) argues, ‘[t]he COVID crisis is suffused with discourses, practices and emotions related to people’s reactions to risk and uncertainty’ (p. 2). Correspondently, our study is broadly situated within the ‘risk society’ framework (Beck, 1992, 1999) in which individuals and societies are increasingly seen as facing incalculable risks due to the intertwined outcomes of modernization, globalization, and environmental crises and required to take action to protect themselves, accordingly. And as succinctly summarized by Nygren and Olofsson (2020), the key pillars of Beck’s risk society thesis are:

(1) the development of new, man-made, mega risks that threaten the existence of humanity on a global scale; (2) globalization with a world risk society; (3) expert dependence: the insensibility and complexity of risk that leave both politicians and the individual dependent on scientific knowledge; (4) individualization: old social structures such as social class are replaced, or at least hidden by, a new political self- fulfilling subject; and (5) risk positions: although social class and other social structures diminish, inequality remains but in the shape of risk positions. (p. 1033)

Our intention, however, is not to apply Beck’s theory of risk society in whole or in detail, but to borrow some of its key arguments to frame the overall discussion and make sense of how participants approach COVID-19 vaccine passports based on perceptions of risk and other intersecting factors. Although dating back to the late 1980s, the risk society thesis has lost none of its relevance and is in fact receiving renewed interest against the backdrop of the current COVID-19 pandemic (Lupton and Willis, 2021: 1; Constantinou, 2021). This is primarily due to the radical uncertainties and myriad disruptions to everyday life brought about by COVID-19, as well as the various mechanisms of pandemic governance that have been proposed/adopted, including vaccine passports, which produce their own set of (secondary) risks. The global aspect and large scale of COVID-19 pandemic also echoes Beck’s risk society thesis, which highlights the fact that modern risks transcend national boundaries taking on a cosmopolitan outlook with far-reaching global consequences (Beck, 1992, 1999). The appeal to scientific expertise and individual responsibility in the ‘fight’ against COVID-19 resonates further with Beck’s thesis as our dependence on experts (e.g. epidemiologists, virologists, and public health professionals) and individualizing techniques of pandemic management (such as quarantine and self-tracking apps) is evident in the current climate. Moreover, COVID-19 and its risks have not been affecting everyone in the same way nor has their impact been felt and experienced equally. For although coronavirus itself has *indiscriminately* struck the entire world creating what Beck’s refers to as the ‘boomerang effect’ (Beck, 1992: 23), the *distribution* of COVID-19 risks seemed to follow the same familiar lines of class and racial divisions, poignantly exemplified by the disproportionate exposure to the virus of those who did not have the option of working from home and were thereby forced to take more risks than others during the pandemic so that the rest remain fed and cared for (Ajana, 2021b: 24). In the United Kingdom, this has led to excess deaths among groups from BAME (Black, Asian and minority ethnic) backgrounds during the early stage of the pandemic, given their overrepresentation in frontline roles and precarious occupations. Such reality both echoes and extends Beck’s (1992) following assertions:

Some people are more affected than others by the distribution and growth of risks, that is, social risk positions spring up. In some of their dimensions these follow the inequalities of class and strata positions, but they bring a fundamentally different distributional logic into play. Risks of modernization sooner or later also strike those who produce or profit from them [. . .]. Simultaneously, risks produce *new international inequalities*. (p. 23)

Inevitably, risk is bound with issues of power, and risk management techniques can often intensify class and racial differences if only as a side effect rather than an intended outcome. As a risk management technique, COVID-19 vaccine passports have sparked many questions around their potential to generate new forms of inequality and power differentials, aggravating all the more the impact of COVID-19. More specifically, there are concerns about the risk of unfairness and discrimination that might become the by-product of establishing a system of triage based on vaccination and immunity status, especially if COVID-19 vaccine passports become mandatory for travel (Ada Lovelace Institute, 2021b; Ajana, 2021a; Brown et al., 2021; Hall and Studdert, 2021a, 2021b; Osama et al., 2021). It is, therefore, questioned how moral it is to establish a system that will privilege significantly those with access to COVID-19 vaccines while disadvantaging low-income populations with poor resources and weak vaccination coverage.

Existing academic literature also picked up on the risk of stigma that might be associated with the possession (or lack thereof) of an immunity/vaccine-based certificate, which can effectively lead to unequal treatment of individuals on the basis of their vaccination status (Bardosh et al., 2022; Mullin, 2020; Persad and Emanuel, 2020; Voo et al., 2020, 2021). According to Hall and Studdert (2020, 2021b), vaccination passport systems have the potential to discriminate against those who refuse vaccination due to philosophical or religious reasons. Davis (2020) cites a number of examples whereby minority groups were targeted and blamed for coronavirus transmission, adding that the deployment of digital contact tracing and vaccination status apps may intensify stigmatization, blame, and even violence toward ethnic and religious groups. For, as Cook et al. (2021) assert, ‘[d]espite the institutionalisation of risk, individuals and populations can be made responsible for safeguarding (or surveilling) themselves and others, revealing that risk perceptions are also individual and relational’ (p. 209). Such issues relate to Beck’s individualization theory whereby he argues that, in the risk society, people are expected to actively seek out information about risks and become agents of their own protection. Those who do not often end up being blamed and stigmatized, as demonstrated in Mary Douglas’ (1992) book, *Risk and Blame*. Referring to the work of both Beck and Douglas, Deborah Lupton (2022) argues that ‘“risks are always political” and judgements of blame and attributions of responsibility are always part of risk politics and cultures’ (p. 90).

The surveillance of individuals and populations has always been an important component of any pandemic management effort. COVID-19, however, has taken surveillance to a different dimension. As a pandemic that has unfolded against the backdrop of an already highly digitalized and datafied world, COVID-19 has seemingly normalized the acceleration and intensification of digital monitoring and control, raising concerns over privacy issues (Beduschi, 2022; Lyon, 2022; Sharma et al., 2022). Italian philosophers, Giorgio Agamben (2020) and Roberto Esposito (2020) both lament the normalization of the state of emergency declared following the outbreak of coronavirus, and the unleashing of various intensive protective measures. They see this as symptomatic of a biopolitical form of governance which brings political procedures of democratic regimes into conformity with those of authoritarian states by sacrificing civil rights and privacy in the name of protecting the population (Esposito, 2020; see also Ajana, 2021b). This impulse is, in fact, inherent to the logic of the risk society, according to Beck. He writes,

As the threat grows, the old priorities melt away, and parallel to that the interventionist policy of the state of emergency grows, drawing its expanded authorities and possibilities for intervention from the threatening condition. Where danger becomes normalcy, it assumes permanent institutional form. (Beck, 1992: 78)

French and Monahan (2020) point out that China’s use of heavy-handed techniques of surveillance-based control to contain COVID-19 has drawn praise from Western sources that might have previously criticized those tactics as breaches of human rights (p. 6). The imperative to protect has turned many surveillance techniques into routine and unquestionable practices, resulting in the extension of governments’ powers and the strengthening of the state’s control over citizens. It is not that such critique denies the necessity of disease surveillance and virus containment. But what it takes issue with is the *entanglement* of public health surveillance and the surveillance of individuals, which creates risks to privacy, data protection, and civil rights. The fact that COVID-19 vaccine passports hold various data about the individual, including vaccination status, raises issues about the possibility of data misuse or repurposing. As Ajana (2021a) argues, we have seen time and again throughout the recent decades how practices and mechanisms that are initially designed for specific exceptional circumstances end up becoming routine and widespread across the entire fabric of society. Biometric technology is a case in point given how this technology is no longer confined to ‘exceptional spaces’ (e.g. airports, prisons, and detention centers), but has become embedded in everyday products and services (e.g. in fitness trackers, smartphones, smart watches, online banking, mobile pay, etc.). With the current technologies being developed to manage people’s health status through biometric apps, there is a risk of a similar function creep and repurposing that may well outlive the pandemic itself (Ajana, 2021a).

Besides such ongoing socio-political concerns, early debates and considerations regarding COVID-19 vaccine passports have largely revolved around the effectiveness of the COVID-19 vaccines themselves. During the development of the vaccines and at the beginning of their rollout, many uncertainties were expressed vis-a-vis their safety and effectiveness, which also called into question the effectiveness of potential vaccination passport systems. For instance, Hall and Studdert (2021a, 2021b) questioned whether vaccine protection would be sufficient against new variants of the virus. Also, even when vaccines proved to significantly decrease infection rates, they cannot completely prevent the transmission of the virus (Harris et al., 2021). Cash-Goldwasse (2020) argues that there are certain assumptions attached to COVID-19 passport systems that scientifically, have not been proven yet. These include (1) vaccination as a proxy for protection, (2) natural infection as a proxy for immunity, and (3) antibodies as a proxy for protection. Based on the current evidence, there is no guarantee that vaccination, natural infection, or antibodies suffice for protection from the virus (Cash-Goldwasse, 2020; Harris et al., 2021). It is argued that there are still many scientific uncertainties surrounding the effectiveness of COVID-19 vaccines, and whether they provide long-term protection and immunity (Brown et al., 2020; Persad and Emanuel, 2020; World Health Organization (WHO), 2021). Furthermore, the lag between the infection and antibodies, that is, the time the body needs to form antibodies after it catches infection, may cause serologic tests to come back falsely negative (Voo et al., 2020). There is, as such, the risk that the implementation of COVID-19 vaccine passports might end up giving a false sense of safety and protection (Ajana, 2021a). For all of these reasons, the *raison d’être* and effectiveness of COVID-19 vaccine passports remains questionable (Brown et al., 2021; Dye and Mills, 2021). Relatedly, it is argued that any viable COVID-19 passport system needs to be dynamic and able to respond to emerging evidence with regard to the vaccines’ efficacy over time and against new variants (Ada Lovelace Institute, 2021c). Anticipation is indeed a key component of the risk society which drives many risk modeling and management techniques (Beck, 1992: 33, 34).

What the above concerns show is that mechanisms of risk management, such as COVID-19 vaccine passports, carry their own risks which, despite the presumed benefits, might in turn affect public perceptions and attitudes toward such techniques and the level of acceptance and compliance vis-a-vis COVID-19 management measures. As Barello et al. (2022) argue, ‘the public following or adhering to health policies or guidelines does not imply an automatic process of acceptance’ (p. 2). Indeed, publics are not just docile objects of government and institutions, but subjects that can comply, resist, adapt, and question the rationalities, motives, and means of governing, even in times of crisis (Foucault, 1977). This depends on and is shaped by myriad factors, including risk perceptions, trust in institutions, and attitudes toward the proposed/deployed measures. Therefore, examining public understanding and perceptions of COVID-19 vaccine passports is important not least given the fact that the public is a key stakeholder that is at once the object and the subject of governance in the risk society (Beck, 1992: 9). This also matters because ‘accountable governments need to be aware of public attitudes if they want to act in a way that takes into consideration the population’s doubts and feelings’ (Barello et al., 2022: 2). Based on this, our study aims to explore how lay people make sense of vaccine passports in relation to their perceptions of risk and how these intersect with values such as equity and trust. While there are many existing studies that address public perceptions and opinions regarding the deployment of COVID-19 vaccine passports (using quantitative survey methods primarily) (Abrams et al., 2021; Ada Lovelace Institute, 2021c; Barello et al., 2022; Hall and Studdert, 2021a; Ipsos, 2021; Lewandowsky et al., 2021), hardly any study has explicitly made the connection between public perceptions of COVID-19 vaccine passports and the risk society theory. We believe such connection can be fruitful in conceptualizing the interplay between these technologies and the public, while contributing to the debates on the socio-ethical implications of vaccine passport systems.

## Study details

The study deployed a qualitative method consisting of online semi-structured interviews with 23 UK-based participants. All UK residents above the age of 18 were eligible to participate in the study. The interviews were conducted during May and June 2021 using Microsoft Teams and Zoom. Prior ethical clearance for this study was obtained from the authors’ institution. The study participants were recruited using social media platforms. Announcements to take part in the study were shared on the authors’ social media handles and within relevant Facebook interest groups such as ‘Covid-19 Research Involvement Group’ and ‘Covid19 vaccine discussion group’. A total of 75 responses was received. Those interested in taking part were asked to fill an online form that collected data in four domains: demographics, education, profession, and COVID-19 vaccination status. Purposive sampling was used for selecting participants from the completed online forms to ensure as much diversity as possible across all domains.

The selected participants were then contacted and invited for the online interview. They were sent an information sheet on the aims, objectives, and methodology of the study as well as the participants’ role and their right to withdraw from the study at any point. All participants gave verbal and written consent to take part in the study and have their interviews recorded and used in subsequent publications. In order to preserve the anonymity of participants, pseudonyms have been used and identifying information has been removed. Some interviews were recorded in audio format and others in video format, depending on the participants’ preferences and what they felt comfortable with. The recorded files were stored on a secure cloud server that is suitable for storing sensitive data in accordance with the Information Security Procedures of the authors’ institution. They were later transcribed by a General Data Protection Regulation (GDPR)-compliant company and analyzed by the authors.

The interview group comprised of 12 females, 10 males, and 1 non-binary participant. In terms of participants’ age, we had a relatively balanced distribution across age groups with 5 participants falling within the 18–25 age category, 7 within the 26–35 category, 4 within the 36–45 category, 2 within the 46–55 category, 5 within the 56–65 category, but no participation from those who are older than 65. This might be due to using online platforms for participants’ recruitment and interviewing. According to a report by the Centre for Ageing Better (2021), 32% of those who have never or not recently used the Internet in the UK were aged between 50 and 69, and 67% were aged 70 or over. This poses a challenge for researchers who wish to conduct online research with participants from these age groups. At the time of interviewing, not all age groups were eligible to receive the COVID-19 vaccine as yet; 4 participants received two doses of COVID-19 vaccine, 8 received the first dose, 9 were awaiting the first dose, and 2 refused the vaccine. Those with postgraduate level of education made up 57% of participants. In terms of ethnicity, more than half of the participants in our study were White British and from other White backgrounds (14), 2 were South Asian, 4 were Arab, 2 from a mixed background, and one participant from Black Caribbean ethnicity. Notably, this participant was the only one from this ethnicity in the initial pool of 75 respondents who replied to our announcements. The low representation of participants from Black minorities might be due to the underrepresentation of such ethnicities within the online COVID-19 interest groups, discussion forums, and circles from which the participants were recruited. This might also have to do with the fact that Black minorities continue to be the group with the highest hesitancy level toward COVID-19 vaccine (Asaria et al., 2021; Kearney et al., 2021; Padamsee et al., 2022), which can also affect their willingness to partake of COVID-19-related research. Although the low representation of Black minorities in our study is, admittedly, a major limitation, we have nonetheless managed to include participants from other underrepresented groups, including Arab and South Asian participants. Further research will seek to recruit a more representative and ethnically diverse sample. Interview questions were guided by a semi-structured approach based on the research questions and the conceptual background mentioned in the previous section. The focus of the study was on exploring participants’ understandings of and attitudes toward COVID-19 vaccines and vaccination passports as well as the factors, such as risk perceptions, influencing these. This article presents the findings pertinent to the vaccine passports, whereas findings pertinent to the vaccines are presented in another article (Ajana et al., 2022). At the time of conducting the interviews, COVID-19 vaccine passports were in the initial state of being introduced in the United Kingdom. As such, participants were first asked whether they have heard of the vaccine passports or not, and if so, whether they know how they would be implemented. They were also asked to elaborate on how they feel about the use of vaccine passports as a means of managing the pandemic going forward. We further explored whether the prospect of traveling more normally using vaccine passports influenced participants’ perceptions and opinions of the passports.

Interview data were systematically codified with the help of NVivo and analyzed in accordance with an interpretative narrative approach (Davis and Lohm, 2020; McQueen and Zimmerman, 2006; Wiles et al., 2005), highlighting the important aspects in the participants’ accounts and placing such narratives in the context of the relevant literature and the risk society thesis. This approach was particularly suited as it allowed a conceptualization of the major themes emerging from the interviews and as experienced and narrated by the participants themselves. This is while ensuring reference to the central objectives of the study.

## Findings

We asked participants to share their views on the introduction of COVID-19 passports for travel and domestic use as well as their potential to manage the pandemic going forward. Notably, not all participants have heard of the COVID-19 vaccine passports prior to seeing the announcement to take part in this study. As mentioned earlier, at the time of conducting our research, COVID-19 passports have just been introduced in England from 17 May 2021, which is partly why some participants were not very familiar with these artifacts as yet. In describing the functionality and purpose of COVID-19 passports to participants, and as articulated by the government and the National Health Services (NHS), we tried to remain as neutral as possible in order not to influence participants’ views on these technologies and obtain responses that are not biased by our own positions. Some participants complained that there has not been enough information on the media and through government communication about COVID-19 vaccine passports prior to their introduction. Some saw this as reflecting the government’s indecisiveness regarding how best to manage the pandemic: ‘I don’t think there’s been a lot of information because I don’t think the government really know what to do, they’re not sure what’s the best action to take’ (Linsey, 49, female, White British, local government office, not willing to get vaccinated). As argued by the Institute for Government (2022), the government has indeed ‘gone back and forth’ on domestic COVID-19 vaccine passports, impacting public confidence in this mechanism and in the whole pandemic management approach, as a result.

It was suggested that the government was using the prospect of mandating COVID-19 vaccine passports in order to push individuals to get vaccinated (Institute for Government, 2022; Woodcock, 2021). One participant did, in fact, decide to get vaccinated,

because there was a lot of talk coming about potentially having vaccination passports and stuff to go abroad or to the pub and stuff. I thought, well if that’s going to be mandatory then I want to get in there quick so that I can go. (Emma, 26, female, White British, student in Psychology, received first dose of vaccine)

Many other participants also considered the introduction of COVID-19 vaccine passports as a way of encouraging people to get vaccinated, especially when travel is concerned:

As far as I know, the government here in the UK and other governments around the world, especially in Europe, now, they are preparing people to have the vaccination passport which will allow them to travel freely between the countries and this counts as encouragement for people to get their vaccines. (Hakim, 26, male, Arab, medical doctor, received first dose at the time of interview)

The prospect of traveling and reuniting with family and friends was, in fact, a major factor which influenced participants’ attitudes toward COVID-19 vaccine passports. As articulated by the following statement:

[M]y wife’s family live in Asia because my wife is Asian… She hasn’t seen her mum… We’ve not been able to travel to South East Asia. We hope maybe next year we might be able to travel… Now as far as I’m concerned COVID passport would allow me the chance to travel say to Spain or to Thailand or wherever it is or to Asia and to minimize the risk of me bringing COVID into their country. (Paul, 63, male, White Irish, former teacher and volunteer Covid-19 vaccinator, received 2 doses of vaccine at the time of interview)

On the contrary, passports and the prospect of traveling more normally may not be the motivation some look for. As is the case of Linsey (49, female, White British, local government office, not willing to get vaccinated), who is not used to ‘going abroad that much anyway’, she stressed that she would be making a stance against having a vaccination passport since she was, in essence, opposed to having ‘something put inside’ her. Her attitude toward vaccine passports correlates with her anti-vaccine stance. Another participant explained that she and her family, as well as many others she knows do not travel regularly, and so a vaccine passport is not something they eagerly look forward to. She further expressed the concern that regulation may differ from one country to another, which would complicate the issue further:

Even if you have a passport here, that depends on what laws they’re laying down in whatever country you’re traveling to, passport or not. Again, it will only take time. You might have a travel passport here, but if you travel to Greece, is it going to be the same conditions when you enter that country because you’ve had a jab? They might still want to quarantine your ass for 10 days. (Erica, 58, female Black Caribbean, newborn hearing screener, not vaccinated and refuses to)

While the incentive to facilitate travel has been a major drive for public acceptance of COVID-19 vaccine passports as well as the acceptance of the vaccines themselves, for Hakim (26, male, Arab, medical doctor, received first dose at the time of interview), the incentive has more to do with social responsibility and protecting the health of others: ‘I’m viewing the vaccine passport as a way to go out of this pandemic in terms of mainly the health of people [. . .]. Whether it is related to economy or related to travel, it does not affect my opinion’. Other participants shared a similar opinion and viewed COVID-19 passports not only in terms of the promise of convenience but more so as a tool of safety and protection. Some believed that a COVID-19 vaccine passport should be a global rule for travel as ‘it’s for your safety and for the safety of everybody else that you’re traveling with’ (John, 56, male, White British, stage manager, received first dose of vaccine). One participant suggested that COVID-19 vaccine passports should be ‘official like the yellow paper cards we had in the past’ (Amil, 25, male, South Asian, researcher, received both doses of the vaccine). For some participants, however, a vaccine passport would not be necessary or useful if everyone was vaccinated: ‘I suppose if the vaccines work, then once the whole world had them, which is some way away, the passports will be irrelevant’ (Edward, 42, male, White British, opera singer, received first dose of vaccine at the time of interview). They therefore emphasized the importance of increasing the vaccine uptake instead of investing in the implementation of vaccine passports.

There were concerns that the imposition of a vaccination passport may risk further divisions in public opinion. Some participants feared that vaccination passports might cause discrimination as people could be categorized into vaccinated and unvaccinated and treated differently based on such labels. Some participants saw in this the risk of creating an atmosphere of coercion where people who did not want to take the vaccine would be forced to take it:

What is the solution for the people that don’t want to do it? Are they going to just go through the same process of quarantining and having Covid swabs or they’re going to not be able to fly at all? Then that means you can’t see your grandmother if she doesn’t want to do it or something like that. If it’s one member of a family, then the whole family can’t fly [. . .]. I think obviously people should be willing to do it, not being forced to do it. (Yana, 39, female, White Other, director, not vaccinated at the time of interview but might)

I think it will add room for creating a new model of discrimination. It will be like the vaccinated and unvaccinated instead of now dealing with racism and prejudice. that will then become a new area for discrimination in our society. So, I don’t support having Covid passports. (Astrid, 33, female, White Other, assistant publisher, not vaccinated at the time of interview but might)

[I]t’s just discrimination if you say that you’re only allowed to travel if you’ve been vaccinated, but we cannot really vaccinate everyone. (Elsa, 25, female, White Other, student in psychology and neuroscience, not vaccinated at the time of interview but might)

Such concerns were even stronger for those who are vaccine hesitant and might feel discriminated against by restrictions in travel and daily life through vaccine passports if they refuse the vaccine. As the following statement indicates:

I’m obviously not happy about it. I’m probably the minority that doesn’t want the vaccine. Already going back to work and people are asking who’s had the vaccine, and I think it’s personal [. . .] and it’s going to cause a lot of problems. Not everybody can have the Covid vaccine either, so we’re discriminating against people. Okay by choice they don’t want it, but there are genuinely people that are unable to have the vaccine because of their health conditions, and that’s not fair. There’s a lot of Black ethnic minority people that choose not to have the vaccine, and again, that’s going to be discriminatory if you start having passports. To think that you can’t go into a place because you’ve not had the vaccine, I think is utterly ridiculous, because at the end of the day, when you’ve had the vaccine, you can still transmit it. You still might not know you’ve got it and transmit it. (Linsey, 49, female, White British, local government officer, not willing to get vaccinated)

One participant believed that the imposition of COVID-19 vaccine passports might, in fact, discourage further those who are vaccine hesitant and those who believe in conspiracy theories from getting vaccinated (Nora, 25, female, Arab, student in Global Health, not vaccinated at the time of interview but might). This could then become counterproductive in terms of increasing trust in the vaccination program and tackling vaccine hesitancy. This view was also shared by participant, Amil (25, male, South Asian, researcher, received both doses of the vaccine), who thought that vaccine passports might make it harder to convince ‘those who don’t want the vaccine, don’t trust the government’ to get vaccinated.

The risk of discrimination on the basis of vaccination status is a concern that was expressed by participants who were not necessarily opposed to having COVID-19 vaccines but were not yet eligible to receiving the vaccine (as mentioned before, at the time of conducting this research, not all age groups had access to the vaccine):

[L]ike everything else in public health, you don’t want to make these things and then discriminate against groups of people that can’t have it. (Amil, 25, male, South Asian, researcher, received both doses of the vaccine)

[E]veryone needs to travel. Of course, these passports will facilitate for people to travel but it will only facilitate for the people who have the vaccines. It will not facilitate the travel of other people who did not get the vaccine. It will be discriminatory. It will be unfair for people who did not get the vaccine. (Yacine, 26, male, Arab, human rights researcher, not vaccinated at the time of interview but might)

As such, a participant explained that COVID-19 vaccine passports should not be implemented until everyone had been offered the vaccine and made a conscious choice about their vaccination status:

This should not be applied until every single one in the country [has] the accessibility and becomes eligible to get the vaccine. (Hakim, 26, male, Arab, medical doctor, received first dose at the time of interview).

Hakim’s stance was not only confined to the case of the United Kingdom but also touched on the global context. He states:

having the vaccine passport and how it could affect the travel worldwide is something I disagree with, because many people around the world have no access to the vaccine, which could hinder their ability to travel into countries which already have the vaccine.

For similar reasons, some of the study participants argued that rich countries have the moral responsibility to donate vaccines to poorer countries and that the rollout of COVID-19 vaccines should be done on an equal global level. More than a moral imperative, vaccine solidarity at the global level is also seen as a pragmatic strategy to reach herd immunity worldwide and bring the pandemic to an end:

Actually, for the wealthy countries, they should take part in providing all the support, the financial support for other countries to get the vaccine either in terms of the vaccine itself or facilitating the administration of it. For me, this will be the only thing that could be done to end the pandemic because it affects everyone in the world, and everyone in the world could be the source of infection to other people because we are open to each other. (Hakim, 26, male, Arab, medical doctor, received first dose at the time of interview)

Participants also expressed concerns regarding the use of COVID-19 vaccine passports and immunity status apps in domestic settings rather than just travel. Since some people are unable to get vaccinated for medical reasons and the fact that some elderly people or those without access to phones may not easily be able to use vaccination passports and immunity status apps, participants saw a risk of discrimination and exclusion arising if such technologies become a requirement for accessing everyday services and public spaces. Speaking of the technical challenges facing her elderly mother, participant, Jenny (48, female, White other, executive assistant, received first dose of vaccine), articulated the issue in the following way:

[M]y mom, for example, she finds incredibly difficult to work out how to use them [apps] [. . .]. The number of times I have to explain to my mom what a QR code is, then which app it is because you’ve got two NHS apps on the phone. Now, why are there two? I don’t understand. Then I’d have to tell her which one because she said she landed the wrong one. Then I have to tell her how to get to the QR bit because she’s standing outside the restaurant with some friends and she can’t get in. She can’t remember how to use the QR code. She doesn’t have Alzheimer’s, she’s 85. She’s just at that age where she needs something that’s really, really simple [. . .]. I can’t say I’m particularly impressed with the apps or the way that they have been rolled out and there’s a long way to go to make them truly accessible to all.

Apart from issues of fairness, discrimination, and technical accessibility, some participants also spoke of their mistrust toward the government, which influenced their attitudes toward COVID-19 vaccine passports. More specifically, participants questioned the government’s ability to securely store data and maintain users’ privacy. They feared a potential leak of health data and other sensitive information as well as the prospect of being tracked:

I wish the government will get a handle on the leakiness of the information that comes from the apps. The other day, it was discovered that the app was leaking information about people’s status and so it is really, really poor. I work for a tech giant and it is really easy to design an app that keeps the information secure. It’s pretty poor that we can’t do that [. . .]. The government hasn’t done a good enough job in that. (Jenny, 48, female, White Other, executive assistant, received first dose of vaccine)

The only concern I would have is the security of any app. It’s health data. What security is there in place? [. . .] Just with any health data, you got to be careful with. That’d be my only concern. (Ted, 26, male, mixed ethnic background, student, not vaccinated at the time of interview but willing to)

I’d say the main concerns are the tracking and knowing where you’ve been. How is that going to be stored and used? Obviously, the government are probably going to be like, ‘Oh well, it will be kept secure and stuff’. You never know, it could be open to hackers to get that information to know where people are. It could be sold to third parties to use the data. Even in research, universities might then have access to it to see how people have been using their passports, etc. (Emma, 26, female, White British, student in psychology, received first dose of vaccine)

While acknowledging the above-mentioned issues and concerns, some participants still believed that COVID-19 vaccine passports should be a component of the plan leading us forward out of the pandemic. For instance, Paul (63, male, White Irish, former teacher and volunteer COVID-19 vaccinator, received 2 doses of vaccine at the time of interview) believed that a vaccine passport system is ‘an important tool in going forward’, especially with regard to the ability to travel. Nathan (26, male, Mixed background, not vaccinated at the time of interview but willing to) also saw vaccine passports as an easier way ‘to not quarantine’ and ‘to live your life’ since it would allow the holder to confirm her vaccination status when traveling to a different country. For Amil (25, male, South Asian, researcher, received both doses of the vaccine), he considers COVID-19 vaccine passports as an important component of pandemic management and a way of encouraging people to get vaccinated and thereby increasing vaccine uptake. He was in favor of requiring vaccine passports to travel and access certain venues like nightclubs where COVID-19 could be easily transmitted. Nevertheless, he did not see vaccination passports as ‘the entire strategy’:

I think it has to be complemented by continuing testing, contact tracing, new therapeutics, that kind of thing. It’s a big component, but not the whole thing [. . .]. Then just building back better and making sure this doesn’t happen in the future, so having more funds to research, more funds for clinical care, increased awareness among people.

Like Hakim’s above argument regarding the need for global equity of vaccine access, Amil also stressed that any plan of managing the pandemic in the future needs to seriously consider this issue of equity: ‘Low-income countries are going to get all these variants, it’s just going to be a self-repeating cycle. That’s the main one, having equitable access to vaccines’. This, by extension, makes vaccine passports a matter of equity too, for Amil.

Edith (25, non-binary, software engineer, not vaccinated at the time of interview but willing to) saw the benefit of COVID-19 vaccine passports in terms of pushing those who are hesitant to get vaccinated:

I think it will push them to get it just to be like, ‘well, we want to go on holiday. We want to get back to normal’. [. . .] It certainly makes sense for making sure everyone coming in and coming out, has had the right doses, or we’re not spreading anyone that hasn’t had a vaccine out to other countries. I’m not sure if I could come up with anything better’.

This is despite her concerns regarding the security of the data that would be collected through digital vaccine passports. For her, it is important that institutions are transparent about where, how and for how long vaccine passports data will be stored and used, and that users are kept informed about how and when relevant databases are maintained and accessed. The success of vaccination passports is, therefore, linked to other factors including data protection and users’ privacy, according to Emily.

## Discussion

This study looked at public perceptions of COVID-19 vaccine passports. The findings highlight that participants’ attitudes toward these passports are primarily driven by factors relating to perceptions of the risks relating to vaccine passports not only for the individual, but also for society. This contradicts the findings of Lewandowsky et al.’s (2021) study, which show that the key driver of acceptance of immunity passports was a variable unrelated to perceptions of risks and harms relating to COVID-19 and immunity passports. For many of our study participants, COVID-19 vaccine passports are also considered as an important component of managing the pandemic going forward, and accelerating economic recovery and return to normality. While this corresponds with the arguments and findings of other studies (Gönüllü et al., 2021; Mbunge et al., 2021; Tsoi et al., 2021), it also highlights a degree of ambivalence in the subjective assessments of COVID-19 vaccine passports. Such ambivalence is primarily due to participants’ awareness of both the potential ability of vaccines, and by extension vaccine passports, to mitigate the risks of COVID-19 and of the risks inherent in the use of vaccine passports themselves. So, rather than being either for or against COVID-19 vaccines passports, many participants demonstrated nuanced views on these technologies with risk being a major aspect that shapes their views. This ‘risk consciousness’ to use Beck’s (1992) term is, thus, far from being one dimensional but can straddle competing narratives and conflicting perceptions that are key to how people tend to weigh the benefits and risks of a given solution (p. 77). In our study, this came through the various themes emerging from participants’ narratives.

First, COVID-19 vaccine passports were considered by many participants as a way of incentivizing people to get vaccinated. Some participants saw this as a positive strategy in terms of the potential of vaccine passports to increase vaccine uptake and thereby contribute to the protection of public health and the reduction of COVID-19-related harms. This positive attitude toward vaccine passports was, for some participants, based on the belief that vaccines themselves are the key tool for managing the pandemic and protecting the population from the transmission of COVID-19 and its more severe effects. In such cases, attitudes toward vaccine passports seemed to correlate with attitudes toward the vaccine. This resonates with the findings of Khan et al.’s (2022) study in which they argue that ‘positive opinions about the usefulness of vaccine passports are predicated on the assumption that vaccinated individuals are protected from severe COVID-19 infection, and they will not spread the virus to others’ (p. 5). Beyond this, such perceptions embody the belief that vaccine passports can demarcate the boundaries between the ‘risky’ and the ‘safe’ based on vaccination status. This echoes Lupton’s (2022) argument that ‘distinctions between Self and Other have continually been made during the COVID crisis, highlighting entanglements between material-discursive concepts of selfhood, the body and more-than-human assemblages’ (p. 133). As a mechanism that links identity to vaccine/immunity status, COVID-19 vaccine passports are indeed a technique of differentiation and identification. They are ‘technologies of the self’ (Foucault, 1988) which serve the ‘individualizing’ aspect of the risk society (Beck, 1992) in which individuals are expected to responsibly monitor their conduct and partake of risk management activities through available technologies. Opting out can itself feel like a risk of its own given the potential exclusion from spaces and services.

Other participants saw the imposition of vaccine passports to encourage vaccine uptake as a coercive strategy that might end up discouraging even more those who are vaccine hesitant. This was also the conclusion of a large-scale national survey conducted by De Figueiredo et al. (2021) in the United Kingdom which found that vaccine passports may result in a lower inclination to accept COVID-19 vaccines. This was more so the case if passports were required for domestic use rather than for international travel, according to the study. A similar sentiment was expressed by some of our study participants who, while not opposed to the use of COVID-19 vaccine passports for international travel, were reluctant when it came to their use in domestic settings. Part of their concern had to do with the risk of vaccine inequity and the fact that some people might not be able to get vaccinated for medical or religious reasons. This led participants to raise the related issue of discrimination which has been at the forefront of the debates on COVID-19 vaccine passports and immunity certificates since their initial proposal. As mentioned earlier in this article, Beck’s risk society thesis draws attention to the fact that the consequences of global risks are not experienced equally, as the socially disadvantaged groups tend to bear the brunt of modern risks more than others. One way in which this has manifested in the context of COVID-19 is indeed the unequal access to COVID-19 vaccines worldwide. Since the beginning of the vaccine rollout, concerns have been raised about how the delivery of COVID-19 vaccine programs could lead to widening health and economic inequalities if adequate steps are not taken, especially in the context of vaccine supply constraints and the prioritization of specific population groups. While the world’s richest countries have secured enough vaccine doses to immunize their populations multiple times over, many poor countries have only been able to immunize a small percentage of their population (Ajana, 2021b). Burgess et al. (2020) state that,

Estimates as of Dec 2, 2020, suggest direct purchase agreements have allowed high-income countries to secure nearly 4 billion confirmed COVID-19 vaccine doses, compared with 2.7 billion secured by upper and lower middle-income countries. Without such agreements, low-income countries would probably have to rely on COVAX, which would achieve only 20% vaccination coverage—which is not enough for achieving herd immunity through vaccination.

More recent data on the share of the population having received at least once COVID-19 vaccine dose by country (KFF, 2022) indicate that low-income countries have only managed to achieve 12% vaccination coverage as of 31 March 2022 in comparison to 77% coverage achieved by upper middle-income countries. This could leave behind populations and communities from already disadvantaged areas, reinforcing health inequalities and worsening the impact of COVID-19. By extension, mandating the use of COVID-19 vaccine passports for travel, without ensuring equitable access to the vaccines and adequate coverage, runs the risk of reinforcing a discriminatory dual regime of circulation. As argued by Ajana (2021a), we are already living in a ‘world apartheid’ whereby the amalgamation of borders, passports, and biometric technologies has been instrumental in creating an international class differentiation through which some nations can move around and access services with ease while others are excluded and made to endure an excess of documentation and securitization. Introducing COVID-19 vaccine passports as ‘tokens of freedom’ will add yet another layer of inequality and discrimination, the consequences of which are likely to outlive the pandemic itself (Ajana, 2021a). Brown et al. (2021) also discuss how vaccine passport holders might enjoy a greater number of privileges during the pandemic (e.g. easier access to travel and leisure facilities), while the civil liberties of non-passport holders remain restricted. Such inequalities could be further deepened if vaccine passport holders will be able to be selected for certain types of jobs which non-vaccinated people cannot have (Ada Lovelace Institute, 2021a). It is thus not surprising that some participants felt ambivalent about the place of COVID-19 vaccine passports in society and globally. For some, the success of such systems rests on ensuring equity in the COVID-19 vaccine coverage worldwide.

Nevertheless, the risk of inequality is not only a matter of vaccine coverage but also relates to technology itself, given the reliance of vaccine passports systems on digital technologies (e.g. biometrics and blockchain) and smartphones. A report by the Ada Lovelace Institute (2022) on COVID-19 technologies, questioned ‘the assumption that technologies will be experienced equally by people’ (Ada Lovelace Institute, 2022: 17), especially that not everyone has equal access to necessary devices and reliable Internet connection, or equal skills to use the technologies effectively (p. 18). There is, in fact, a high degree of technological determinism dominating the debates on vaccine passports, which seems to ignore that the digital divide still exists in the world. And here the digital divide is not just about those who own and those who do not own smartphones, but also relates to what smartphone model one has. Already the deployment of COVID-19 contact tracing apps has revealed the flaws of digital solutionism, as the majority of COVID-19 apps are not operational on older models of smartphones nor on phones that do not support Android or Apple operating systems. As stated on the UK Health Security Agency (2022) website:

The app does not work on:

- Apple phones using lower than iOS 13.5 (includes iPhone 6, 6 Plus and earlier models).
- Android phones using lower than version 6.0 (Marshmallow).
- Windows phones launched before May 2019.
- Huawei phones launched from May 2019.

As such, technological affordances also play a role in shaping access and experiences of COVID-19-related apps and can lead to forms of exclusion as a by-product risk. The scope of this exclusion can also manifest in embodied aspects related to disabilities, as the design of mainstream smartphones and applications tend to be calibrated on the ‘healthy bodies’, ignoring, for instance, people with weak eyesight or hearing who cannot discern many digital signals. Our study participants were indeed mindful of the technical challenges facing their elderly relatives as they navigate the COVID-19 cyberworld. The individualization dimension of the risk society of which Beck speaks is continuously haunted by the specter of exclusion, since it is built on the assumption of technological literacy and ability to proactively be in charge of one’s self-care and risk management. But those who lack such form of literacy and competencies risk being left behind. Furthermore, the imposition of digital vaccine passports also seems to ignore that some people do not wish to have their everyday activities completely dependent on a digital app or a certificate. The right to be ‘disconnected’ from the digital datafied world, as hard as it is in today’s contemporary setting, should still be respected and protected (Ajana, 2021a).

The risk of exclusion and discrimination might also result from data being used for profiling purposes, targeting those identified as having greater vulnerability to COVID-19 due to their pre-existing health conditions or vaccination status. The report by Ada Lovelace Institute (2022) touches on this point, arguing that technological interventions, such as those of vaccine passports, could mean that ‘the link between health inequalities and social inequalities is exacerbated’ (p. 19). This indeed emerged as a major worry in the responses of our study participants, some of whom took issue with the risk of data-driven forms of unfairness and discrimination not only for the UK society but at a global level, given the danger of creating a two-tier system that favors those who are fully vaccinated and in possession of vaccine passports (see also Meaney, 2021; Voigt et al., 2021; WHO, 2021). Discrimination might also become the by-product of the lack of standardized regulations across the world concerning vaccine passports and other COVID-19 technologies, as expressed by one participant. For such reason, Wang and Ping (2022) argue for bilateral and multilateral accreditation of vaccination certificates to ensure unified standards across countries (p. 760). In the risk society, however, adequate regulations are often insufficiently developed (Beck, 1992: 42) given the speed by which technological solutionism proceeds and the slow development of legal frameworks in comparison. This is more so the case of risks that carry a sense of urgency, such as COVID-19, as they tend to induce a need for quick tech fixes leaving no time for the relevant laws and policies to be developed and implemented.

Correlatively, participants were aware of issues of tracking and surveillance as well as data misuse and repurposing that could be intensified through the deployment of COVID-19 vaccine passports. Other studies have also raised similar issues calling for more robust policies around data collection, sharing, and use in light of the increasing reliance on digital technologies to manage the COVID-19 pandemic (Tsoi et al., 2021; Wang and Ping, 2022). Renieris (2021), for instance, argues that ‘[i]f 9/11 ushered in an era of mass surveillance, the pandemic has the potential to introduce the ‘ID turn’ or the age of ubiquitous identification and the end of anonymity’. This shift, according to the author, may outlive the pandemic itself by embedding ‘permanent digital identity infrastructure without our full consideration of the consequences’ (Renieris, 2021). As mentioned before, many scholars such as Agamben (2008, 2020) and Ajana (2013, 2021a, 2021b) have long warned about the function creep of surveillance technologies and the way mechanisms that were initially designed for exceptional circumstances become normalized, widespread, and permanent. Some even take a somewhat abolitionist stance toward digital monitoring technologies given the risks they pose toward human rights and individual freedoms. In our study, some participants do not necessarily oppose the introduction of COVID-19 vaccine passports altogether, as they see this as throwing the proverbial baby out with the bathwater. Instead, they believe that, with adequate policies around privacy and data protection, COVID-19 vaccine passports can fulfill their purpose without compromising users’ civil rights. This is indeed an important counterpoint to some of the debates on COVID-19 technologies which often cast privacy and data protection rights as elements that need to be sacrificed for health and safety, instead of seeing them as equally crucial to the success of COVID-19 management systems (including passports) and societal wellbeing. For instance, at the beginning of the pandemic, the European Data Protection Supervisor, Wojciech Wiewiórowski (2020), argued for the need to adapt existing regulations to make room for more surveillance in order to tackle COVID-19.

However, weighing or rather pitting fundamental rights against security and safety can lead to increasing public mistrust toward institutions and resistance against the mechanisms deployed to contain COVID-19, as evidenced in various studies (Barriga et al., 2020; Jennings et al., 2021; Keshet, 2020; Surber, 2022). For such reasons, Ioannou and Tussyadiah (2021) argue that technological solutions to health crises ‘will only be effective if certain considerations and actions towards respecting individual privacy are also undertaken’, an argument that resonates with many of our study participants (p. 10).

Issues of trust and transparency were also often mentioned in participants’ narratives, especially when discussing privacy, information security, and data protection aspects. For many participants, acceptance of COVID-19 vaccine passports was contingent on the government’s ability to safely and securely handle and store the generated data, and on private companies not misusing or commercializing the data. Other studies have also discussed the role of trust in public acceptance of vaccine passports and the need for transparency in terms of how, why, and for how long such systems would be deployed, and in terms of users’ rights (Ada Lovelace Institute, 2022; Lewandowsky et al., 2021; Vergara et al., 2021). In our study, levels of trust seem to have also been influenced by the perceived competence (or lack thereof) of political leaders. While the majority of participants expressed a relatively high level of trust in scientists and healthcare institutions, they were, however, dissatisfied with the government’s handling of the pandemic, especially at the beginning. Participants described the government’s strategies toward COVID-19, including the deployment of vaccine passports, as ‘indecisive’ and lacking adequate communication with the public. They also took issue with the way the government seemingly underplayed the risks of COVID-19 at the start of the pandemic and favored the herd immunity approach before viable vaccines were available. Trust, in this sense, does not only link to perceptions of the technology itself and its risks, but also of other actors, of their past decisions, responses, and actions. In the risk society, issues of trust and its offshoot notions of credibility, reliability, and transparency are indeed frequently raised (Beck, 1992; Giddens, 1990). While social reliance on risk managers and knowledge experts increases during times of crisis, this does not necessarily translate into an increase in public trust toward such experts and governing institutions. For, as Doyle (2007) argues following from Giddens, ‘expert knowledge is never definitive, never final, always incomplete, imperfect and fluid: this makes it difficult for people to have trust in experts’ (p. 10). What is more, the lack of collaboration between experts and lay people together with the absence of clear governmental and health communication can all contribute to a trust deficit. Beck (1992) highlights similar issues in the following way:

[. . .] what becomes clear in risk discussions are the fissures and gaps between *scientific* and *social* rationality in dealing with the hazardous potential of civilization. The two sides talk past each other. Social movements raise questions that are not answered by risk technicians at all, and the technicians answer questions which miss the point of what was really asked and what feeds public anxiety. (p. 30)

But the decline of public trust is not all too negative. It is also symptomatic of the increased ‘reflexivity’ that is another hallmark of the risk society (Beck, 1992: 99) – the fact that people are more critical and politically aware, more proactive and less trusting of conventional politics (Doyle, 2007: 11). In sharing their risk perceptions toward COVID-19 vaccine passports, our study participants have indeed shown a great deal of reflexivity and the capacity to critically approach governmental responses and technological solutions toward the pandemic. They have shown the inclination to seek out information for themselves from multiple sources, including the Internet. Of course, the risks of misinformation and conspiracy theories loom large in the digital age and can negatively affect public trust and derail risk prevention measures. Creating opportunities and platforms for interactions and collaborations between experts and publics can help ensuring that everyone benefits from the heightened reflexivity of the risk society all the while keeping the risks of misinformation at bay. For as Beck (1992) puts it, ‘scientific rationality without social rationality remains *empty*, but social rationality without scientific rationality remains *blind*!’ (p. 30).

## Conclusion

In this article, we drew on an interview-based study with members of the UK public as well as the relevant literature to examine perceptions of COVID-19 vaccine passports and the extent to which risk thinking or ‘risk consciousness’, as Beck (1992) puts it, shapes such perceptions. We framed our overall discussion within the risk society thesis, conceptualizing the ways in which vaccine passports end up producing their own risks in their attempt to contain and manage the risks of COVID-19 itself. What our findings and those of other studies demonstrate is the need for more open communication and public debates on COVID-19 vaccine passports to enable a more informed civic understanding of the purpose, functions, and implications of such technologies, and more robust assessments of their benefits and risks by multiple stakeholders, including the public. This will help diversifying perspectives on vaccine passports and counteracting the promotional narratives of the tech industry. The findings also demonstrate a stronger need for ethical justifications, mitigation strategies, and appropriate regulations that can preempt the risks of marginalization, inequity, discrimination, and data misuse that can result out of deploying COVID-19 vaccine passports without due consideration of their far-reaching implications. Taking a coercive approach, as the human rights organization Liberty (2021), warns in the context of COVID-19 vaccine passports will undermine relationships and induce distrust and division. While some critics are against the use of vaccine passports altogether and in any circumstances, others are calling for better governance through more open and transparent communication with the public; clearer parameters for use, application, and context; and stricter regulations around data protection and user privacy (Ada Lovelace Institute, 2022). As coronavirus still circulates and concerns over the potential outbreak of other infectious diseases, such as monkeypox, grow, mandatory vaccine passports and health certificates might end up being revived again. This will call for more policy debates and public engagement to ensure the benefits of such systems are distributed equally and their risks are mitigated effectively.
